# Health Information Technology to Facilitate Communication Involving Health Care Providers, Caregivers, and Pediatric Patients: A Scoping Review

**DOI:** 10.2196/jmir.1390

**Published:** 2010-06-18

**Authors:** Stephen James Gentles, Cynthia Lokker, K Ann McKibbon

**Affiliations:** ^1^Department of Clinical Epidemiology and BiostatisticsMcMaster UniversityHamiltonCanada

**Keywords:** Infant, child, adolescent, physician patient relations, communication, medical informatics, applications, computers, Internet

## Abstract

**Background:**

Pediatric patients with health conditions requiring follow-up typically depend on a caregiver to mediate at least part of the necessary two-way communication with health care providers on their behalf. Health information technology (HIT) and its subset, information communication technology (ICT), are increasingly being applied to facilitate communication between health care provider and caregiver in these situations. Awareness of the extent and nature of published research involving HIT interventions used in this way is currently lacking.

**Objective:**

This scoping review was designed to map the health literature about HIT used to facilitate communication involving health care providers and caregivers (who are usually family members) of pediatric patients with health conditions requiring follow-up.

**Methods:**

Terms relating to care delivery, information technology, and pediatrics were combined to search MEDLINE, EMBASE, and CINAHL for the years 1996 to 2008. Eligible studies were selected after three rounds of duplicate screening in which all authors participated. Data regarding patient, caregiver, health care provider, HIT intervention, outcomes studied, and study design were extracted and maintained in a Microsoft Access database. Stage of research was categorized using the UK’s Medical Research Council (MRC) framework for developing and evaluating complex interventions. Quantitative and qualitative descriptive summaries are presented.

**Results:**

We included 104 eligible studies (112 articles) conducted in 17 different countries and representing 30 different health conditions. The most common conditions were asthma, type 1 diabetes, special needs, and psychiatric disorder. Most studies (88, 85%) included children 2 to 12 years of age, and 73 (71%) involved home care settings. Health care providers operated in hospital settings in 96 (92%) of the studies. Interventions featured 12 modes of communication (eg, Internet, intranets, telephone, video conferencing, email, short message service [SMS], and manual downloading of information) used to facilitate 15 categories of functions (eg, support, medication management, education, and monitoring). Numerous patient, caregiver, and health care relevant outcomes have been measured. Most outcomes concerned satisfaction, use, usability, feasibility, and resource use, although behavior changes and quality of life were also reported. Most studies (57 studies, 55%) were pilot phase, with a lesser proportion of development phase (24 studies, 23%) and evaluation phase (11 studies, 11%) studies. HIT interventions addressed several recurring themes in this review: establishing continuity of care, addressing time constraints, and bridging geographical barriers.

**Conclusions:**

HIT used in pediatric care involving caregivers has been implemented differently in a range of disease settings, with varying needs influencing the function, form and synchronicity of information transfer. Although some authors have followed a phased approach to development, evaluation and implementation, a greater emphasis on methodological standards such as the MRC guidance for complex interventions would produce more fruitful programs of development and more useful evaluations in the future. This review will be especially helpful to those deciding on areas where further development or research into HIT for this purpose may be warranted.

## Introduction

The US Institute of Medicine (IOM) has produced several important documents that have had substantial influence on US health care. One of these documents, titled *Crossing the Quality Chasm: A New Health System for the 21st Century,* posits that redesign of the health care process by administrators, health professionals, and patients is needed. The report lays out ten rules with which these players should work. The first of these rules is:

Patients should receive care whenever they need it and in many forms, not just face-to-face visits. This rule implies that the health care system should be responsive at all times (24 hours a day, every day) and that access to care should be provided over the Internet, by telephone, and by other means in addition to face-to-face visits [1].

Pediatric patients with health conditions requiring follow-up and their caregivers (unpaid, including family members and school personnel) is probably the group that can most benefit from what the IOM calls the "continuous healing relationship." Children in need of ongoing medical care are typically dependent on a caregiver to mediate at least part of the necessary two-way communication with health care providers. Many of the common chronic diseases in children, such as asthma and type 1 diabetes, can deteriorate rapidly and have serious complications. Parents or other caregivers must rely on observations and intuition to assess when more or different care is needed or if a health care provider's attention must be sought. Information gathering and transmission are vitally important to parents whose children require care and oversight from pediatricians and primary care providers. The needs of all involved in the care of pediatric patients have been supported in various ways by health information technology (HIT). HIT is increasingly being used and studied for its role in information transfer and health care delivery for pediatric patients in community and home care settings, often with involvement of parents and other caregivers.

The Robert Woods Johnson Foundation described HIT as "the use of a variety of electronic methods for managing information about the health and medical care of individuals and groups of patients [2]." Chaudhry and colleagues, in a seminal review of the evidence supporting HIT, showed that HIT can improve the quality and delivery of care although much research remains to be done, especially in specific disciplines and outpatient and home settings [3].

An important subset of HIT includes applications used for communication between people, often patients or caregivers and health care providers. This subset of HIT is sometimes termed “information communication technology” or ICT. ICT is ubiquitous, and its place in daily lives is growing. One major segment of ICT is in health and wellness. Health ICT can be a simple web page or text message to report blood glucose levels. It can also be complex gene analyses to predict future health in newborns, national electronic health records systems, or automatic international outbreak data gathering and reporting mechanisms. ICT function can be data gathering and analyses, monitoring and alerting (eg, breathing monitors in premature infants), diagnosis and treatment at distances (eg, teledermatogy, telesurgery, or telepsychiatry), or communication. In pediatrics, this communication function is especially important in a context where children with health care needs require caregiver mediation for management of their care.

Because the term ICT is rarely used in the HIT literature currently and was not used in any of the studies in this review, we have opted to use the more general term, HIT, in the rest of this paper. We describe ICT as a separate subset of HIT because we feel this term will be adopted more frequently in the future, especially to describe studies such as the ones included in this review.

Awareness of the extent and nature of published research involving such interventions (henceforth HIT) is currently lacking. We conducted a scoping study with the objective of mapping the health literature about HIT used to facilitate communication involving health care providers and caregivers of pediatric patients with health conditions that require follow-up.

The term “scoping study” can refer to a broad range of activities and has so far only been defined imprecisely. Mays and colleagues proposed that, “scoping studies aim to map *rapidly* the key concepts underpinning a research area and the main sources and types of evidence available [4].” This type of study has also been characterized as a form of literature review that differs from systematic reviews because it “tends to address broader topics where many different study designs might be applicable [5].” More recently, Anderson and colleagues developed the concept further by illustrating the different elements or categories of activity that scoping studies could engender. These include literature mapping, conceptual mapping, and policy mapping. According to their categorization, the current study qualifies as a literature map “designed to provide an initial indication of the size and location of the literature relating to a particular topic as a prelude to a comprehensive review of the literature [6].”

## Methods

The methods for this scoping review were guided by standard review methods and those described by Arksey and O’Malley [5]. Iterative decisions about data collection, fields for extraction, analysis, and so on, were discussed in meetings attended by the authors and documented in a study log.

### Search and Selection

This review was restricted to primary studies of HIT applications used in pediatric care to support communication that involved patients’ caregivers and health care providers.

Searches were informed by 6 seed articles [7-12] and other published searches in relevant reviews of HIT [13-15]. The search approach combined terms relating to the concepts of care delivery, information technology, and pediatrics (Multimedia Appendix 1). MEDLINE, EMBASE, and CINAHL databases were searched on January 22, 2008 and again on February 2, 2009 for articles published between 1996 and 2008. The search was limited to studies in English and excluded letters, editorials, and news items.

Inclusion and exclusion criteria for this complex topic were developed and applied iteratively over three rounds of duplicate screening involving all authors (Table 1). In the first round, titles and abstracts were screened inclusively to retain any articles featuring communication, information technology, and pediatrics. In the second round, abstracts and full text of the articles were reviewed to determine whether electronic technology (including telephone) was used to facilitate communication, and whether there was communication of some sort involving caregivers and health care providers. The third screen occurred during data extraction, when each additional criterion was applied iteratively to the retained set of articles. Publications that studied the same intervention in the same set of patients were matched and classified as a single study.

**Table 1:** Iterative eligibility criteria

	Exclusion Criteria	Inclusion Criteria
First screen	Telephone or email was used for survey or trial recruitment purposes	Electronic health records that allow access by caregivers
	Acute diseases and other conditions not requiring follow-up, including vaccinations	Patient or caregiver use of HIT in settings other than the home, including emergency departments (EDs) or health care provider offices
	HIT used for epidemiological or public health purposes	
	Telemedicine applications where communication was entirely among health care providers	
	Prenatal patients	
Second screen	No communication that involved both caregiver and health care provider	Telephone triage services
	No electronic technology used to communicate	Computer kiosks in health care settings
	Communication while parties were face-to-face	
Third screen	Telephone triage services not explicitly dedicated to chronic diseases or conditions requiring follow-up	Studies of healthy patients, provided the HIT intervention was intended for chronic disease
	Large programs of which telephone was only a small element	

### Data Extraction

Microsoft Access was used to develop a form for data extraction. Initial fields and their definitions were developed and recorded in an accompanying guide based on 6 seed articles [7-12] and a sample of 30 abstracts of articles included in the first round of screening. Data regarding the patient, caregiver, health care provider, HIT intervention, outcomes studied, and study design were extracted from the full text (by SG) and maintained in a Microsoft Access database.

To help summarize the heterogeneity in the study types, we used the framework proposed in the UK’s Medical Research Council (MRC) guidance for developing and evaluating complex interventions [16]. The majority of studies in this review were of complex interventions, defined by the guidance as those with several interacting components and several possible features that make them complex. According to MRC, these features include:

- Number of and interactions between components within the experimental and control interventions
- Number and difficulty of behaviors required by those delivering or receiving the intervention
- Number of groups or organizational levels targeted by the intervention
- Number and variability of outcomes
- Degree of flexibility or tailoring of the intervention permitted [16]

The MRC framework consists of a continuum of four research phases, which may be non-linear: development, feasibility and piloting, evaluation, and implementation. The guidance stresses the importance of reporting of all stages of research and cautions against focusing too heavily on the evaluation phase while neglecting the others. We categorized each study into one of these phases to give an estimate of how each one is represented in this area of HIT research. Definitions of each phase were developed iteratively to fit the studies we categorized in this review while remaining as consistent with the original MRC definitions as possible (Table 2).

**Table 2:** Definitions of the research phases adapted from the MRC guideline for complex interventions [16] used to classify studies

Research Phase	Definition
Development phase	Studies in the development phase are those that investigate intervention design-related outcomes (satisfaction, feasibility, usability) before the intervention has reached a deployable state of development. Also included are theoretical and modeling studies or reports limited to describing the technology or user interactions with it.
Piloting phase	Studies in the piloting phase are those that investigate intervention design-related outcomes when it is a question of refining the intervention after it has reached a relatively complete stage of development. User-related outcomes (behavior change, resource use, clinical outcomes, quality of life) are often measured in the same study. Feasibility and pilot studies that feature user-related measures are differentiated from full-scale evaluations (below) if their outcomes are less important (eg, process outcomes), sample size is small, or a less rigorous study design is used. Some studies reported the adaptation of an existing technology (eg, video-conferencing for telemedicine) for a particular disease, using a case study format where patient outcomes are described. Although these studies do not involve a program of development, they were categorized as feasibility and piloting studies because they report user-related outcomes.
Evaluation phase	Studies in the evaluation phase are those that evaluate important user-related outcomes that use one of the more rigorous available study design options and have a large sample size.
Implementation phase	Studies in the implementation phase are those that evaluate user-related outcomes for an intervention that is well established (eg, in use for more than 2 years) or for which a full-scale evaluation has been published. As many implementation efforts are not reported, it was expected that this phase would have low representation.

### Analysis

Queries were run in Microsoft Access to summarize the data quantitatively. Also, a qualitative descriptive approach was used to summarize how HIT was used and studied in the four most highly represented disease contexts in our study.

## Results

### Study Characteristics

**Figure 1:**
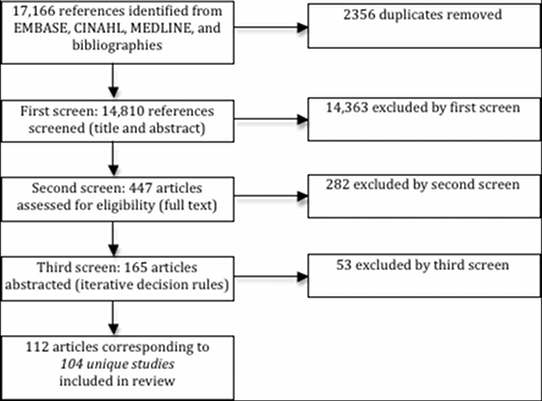
Search and screening results

We identified 104 studies (112 articles) eligible for inclusion (Figure 1). Represented are 30 different health conditions, with asthma, diabetes, special needs and mental health being the most common. Although 17 countries are represented, the majority of studies were conducted in the United States (Table 3).

**Table 3:** Proportional distribution (percent) of studies by disease and country (N = 104)

	Total	United States	Australia	Canada	United Kingdom	Italy	Other^b^
	(n) %	(n) %	(n) %	(n) %	(n) %	(n) %	(n) %
	(104) 100	(53) 51	(15) 14	(12) 12	(6) 6	(4) 4	(14) 13
Asthma [8,9,17-32]	(18) 17	(12) 12	(2) 2	-	-	-	(4) 4
Type 1 diabetes [33-43]	(12) 12	(6) 6	-	-	(1) 1	(1) 1	(4) 4
Special needs [44-55]	(11) 11	(7) 7	(1) 1	-	(1) 1	(1) 1	(1) 1
Psychiatric disorder [56-67]	(10) 10	(4) 4	(1) 1	(2) 2	1	-	-
Various diseases [68-74]	(7) 7	(5) 5	(1) 1	-	-	-	(1) 1
Cancer [75-79]	(5) 5	(2) 2	(3) 3	-	-	-	-
Cardiac disorder [80-84]	(4) 4	-	-	(1) 1	(3) 3	-	-
Sudden infant death syndrome risk [85-88]	(4) 4	(3) 3	-	-	-	-	(1) 1
Burns [89-91]	(3) 3	-	(2) 2	-	-	-	(1) 1
Complex health care needs post-discharge [92-94]	(3) 3	-	-	(3) 3	-	-	-
Speech-language pathology [95-97]	(3) 3	(1) 1	(1) 1	(1) 1	-	-	-
Chronic kidney disease (dialysis) [98,99]	(2) 2	-	-	-	-	(2) 2	-
Cystic fibrosis [100,101]	(2) 2	(1) 1	(1) 1	-	-	-	-
Epilepsy [102,103]	(2) 2	(2) 2	-	-	-	-	-
Traumatic brain injury [10,104-107]	(2) 2	(2) 2	-	-	-	-	-
Very low birth weight [108,109]	(2) 2	(2) 2	-	-	-	-	-
Other^a^	(14) 13	(6) 6	(1) 1	(5) 5	-	-	(2) 2

^a^ Diseases that were the topic of only 1 study that met the inclusion criteria: Anorexia nervosa (Canada) [110], endocrine (Australia) [111], feeding disorders (United States) [112], gastroenterological (United States) [113], hemophilia (Canada) [114], HIV (United States) [115], hypertension (Greece) [116], medical and surgical problems (Canada) [117], recurrent pain (Canada) [118], respiratory failure (Japan) [119], rheumatological disease (United States) [7], scoliosis (Canada) [120], sickle cell anemia (United States) [121], vascular infusion (United States) [122].

^b^ Countries from which only 1 or 2 studies met the inclusion criteria: Germany (2; SIDS, diabetes), Netherlands (2; asthma), Norway (2; burns, diabetes), France (1; diabetes), Greece (1; hypertension), Ireland (1; special needs), Israel (1; asthma), Japan (1; respiratory failure), Multiple (1; type 1 diabetes), Spain (1; various), Taiwan (1; asthma).

#### Participants

Of the 104 included studies, 88 (85%) included non-infant children (2 to 12 years of age), while 94 (90%) included children or adolescents (2 to18 years of age). Adults were also included in 5 (5%) of the studies. Caregivers consisted of family members (generally parents) in 102 (98%) of the studies and included school personnel in 7 (7%) of the studies. Characteristics of study patients, providers, and settings are shown in Table 4.

**Table 4:** Percent of studies with selected participant characteristics (N = 104)

Characteristic		(n) %
**Patient ages**		
	0-24 months	(41) 39
	2-6 years	(63) 61
	6-12 years	(83) 80
	13-18 years	(70) 67
**Patient settings**		
	Home	(74) 71
	Community^a^	(11) 11
	Clinical	(29) 28
**Types of health care provider**		
	Nurse	(38) 37
	Therapist^b^	(25) 24
	Primary care physician	(19) 18
	Sub-specialist	(65) 63
**Health care provider settings**		
	Public health	(3) 3
	Primary care	(10) 10
	Hospital^c^	(96) 92
	Other	(2) 2

^a^ Community settings include school or daycare.

^b^ Therapists include psychologists or counselors.

^c^ Hospital settings include specialty clinics; other settings include call centers or home care.

#### Interventions

Interventions featured synchronous (immediate) transfer of data in 44 (42%) of the studies and asynchronous (store-and-forward) transfer in 36 (35%) of the studies, while in 24 (23%) of the studies, the intervention featured both. Communication commonly occurred via the Internet, telephone, videoconference, or email. HIT function was classified into 15 categories centered on support, medication management, diagnosis, education, and monitoring. Shown in Table 5 are these and other characteristics of the interventions featured in the studies.

**Table 5:** Percent of studies with selected intervention characteristics (N = 104)

Intervention Characteristic		(n) %
**Communication modes featured by HIT intervention**		
	Internet^a^	(34) 33
	Intranet^a^	(6) 6
	Telephone	(26) 25
	Video conference	(46) 44
	Email	(22) 21
	SMS	(3) 3
	Manual download	(13) 13
**Types of data delivered by HIT intervention**		
	Text	(36) 35
	Voice	(53) 51
	Video or imaging	(50) 48
	Multimedia	(18) 17
	Binary	(30) 29
**Functions served by HIT intervention**		
	Caregiver psychological support	(34) 33
	Patient psychological support	(17) 16
	Physiological monitoring	(40) 38
	Behavioral surveillance	(16) 15
	Diagnosis	(36) 35
	Medication management	(49) 47
	Physical care management	(18) 17
	Patient behavior management	(33) 32
	Professional counseling	(33) 32
	Medical consultation	(47) 45
	Mental health tx (non-counseling)	(15) 14
	Education	(41) 39
	Referral	(13) 13
	Transfer patient data to family	(16) 15
	Virtual family visits	(4) 4

^a^ Internet and intranet modes generally excluded telephone, video conference, and email.

#### Outcomes

Of the 104 studies, 72 (69%) measured patient outcomes, 85 (82%) measured caregiver outcomes, 41 (39%) measured provider outcomes, and 58 (56%) measured outcomes at the overall program level. Overall, 86 (83%) of studies measured one of the user outcomes: satisfaction, feasibility, or usability. Of these, 43 (41%) were from the patient perspective, 70 (67%) were from the caregiver perspective, and 34 (33%) were from the provider perspective. Outcomes related to resource use (by patients, caregivers, providers, or the overall program) were measured in 34 (33%) of the studies. Shown in Table 6 are these outcomes broken down by specific outcome categories.

**Table 6:** Percent of studies measuring selected outcomes (N = 104)

Type of Outcome		Overall	Patient	Caregiver	Health Care Provider	Program Level
		(n) %	(n) %	(n) %	(n) %	(n) %
**Broadly applicable outcomes**						
	Satisfaction	(60) 58	(33) 32	(58) 56	(19) 18	-
	Feasibility	(70) 67	(20) 19	(34) 33	(23) 22	(45) 43
	Usability	(39) 38	(23) 22	(35) 34	(14) 13	-
	Usage	(21) 20	(9) 9	(16) 15	(6) 6	(8) 8
	Behavior change	(24) 23	(18) 17	(16) 15	(5) 5	-
	Resource use	(26) 25	(18) 17	(8) 8	(5) 5	(12) 12
**Patient- and caregiver-specific outcomes**						
	Knowledge	(10) 10	(9) 9	(10) 10	-	-
	Clinical outcomes	(33) 32	(31) 30	(2) 2	-	-
	Quality of life	(21) 20	(17) 16	(13) 13	-	-

#### Study Design

Of all studies, 29 (28%) had a qualitative component. Mixed methods were used in 8 (8%) of the studies. The rest were quantitative studies: 17 (16%) of these were randomized controlled trials; 11 (11%) were non-randomized controlled trials; 61 (59%) were descriptive studies; and 7 (7%) were before-and-after studies. Ninety-seven studies (93%) featured complex interventions according the MRC definition [16], while the remaining 7 (7%) were diagnostic studies that did not fit the MRC framework. Using the MRC framework, 24 studies (23%) were categorized as development phase, 57 (55%) as pilot phase, 11 (11%) as evaluation phase, and 5 (5%) as implementation phase.

### Qualitative Themes

HIT interventions were applied to several common problems in the context of pediatric care requiring communication involving caregivers and health care providers: establishing continuity of care, addressing health care provider time constraints, and bridging geographical barriers (Table 7). At least one of these themes was represented in each included study; examples of these are described below for the four most common disease contexts. These sections describe what forms HIT interventions took and how they were studied in each disease.

**Table 7:** Common themes or problems addressed by HIT interventions

Theme	Description	Example Disease Contexts
Establishing continuity of care	Extending care to patients in the community (home, school) beyond settings where they traditionally access care (eg, hospitals)	Complex health care needs post-discharge from hospital
Addressing time constraints	Increasing efficiency of care or reducing time burden on health care providers	ED decision support for asthma
Bridging geographical barriers	Reducing the need for patient travel or providing access to distant specialists	Burn care to patients in rural Australia

#### Asthma

Studies that involved pediatric patients with asthma had the highest representation with 17 studies. Parents of pediatric asthma patients may be asked to keep diaries to monitor use of rescue medication and home spirometry tests (measuring lung function in terms of peak expiratory flow). The health care provider traditionally relies on such manually recorded information to guide patient management. In 8 (47%) of these studies, spirometry was electronically monitored, while in 4 (24%), medication use was electronically monitored. Electronic monitoring was used in these cases (together representing 9 [53%] of the asthma studies) to reduce the burden of paper diary keeping and increase the reliability of the data.

Another common function for HIT in the asthma setting is education, the subject of 9 (53%) of these studies. Studies involving patients with asthma generally featured guideline-recommended information including environmental factors, medications (eg, inhaler use), handling of asthma attacks or emergencies, and the patient’s individual care plan. Monitoring and education were combined in the same intervention in five studies [8,17,20,25,28]. The common goal of including both functions was to establish continuity of care, an important element of managing chronic diseases and one of three recurring themes addressed by the HIT interventions we report on here (Table 7). In 15 (88%) of the asthma studies, data transfer was asynchronous only, reflecting the unique communication needs in this setting.

A dominant function of HIT in studies involving patients with asthma was to improve medication management (14 studies, 82%), a critical step in optimizing disease control and reducing the likelihood and number of attacks that require medical attention. We found 3 studies that featured computer kiosks used for initial assessments, one in a general practice setting [24] and two others in an ED setting [18,21]. Such use of kiosks was unique to asthma among the 104 included studies. In all cases, the intervention was intended to increase the time-efficiency and comprehensiveness of information transfer to health care providers for decision support purposes. These, and other studies that featured educational functions for HIT, provide examples of efforts to address health care provider time constraints, another of the recurring themes observed in this study (Table 7).

We found 12 (71%) studies that succeeded in measuring clinical outcomes (including lung function, symptom control, and use of rescue therapy). Resource use (usually hospital or ED visits) was also commonly measured (10 studies, 59%). A comparatively high proportion of studies that focused on patients with asthma were evaluation studies (7 studies, 41%). Of these, 3 (18%) were development phase, 6 (35%) were pilot phase, and 2 (12%) were diagnosis studies.

#### Type 1 Diabetes

We retrieved 12 studies dealing with pediatric patients with type 1 diabetes. Behaviors underlying medication adherence are traditionally important challenges to, and targets of, management [123]. Correspondingly, both behavioral management (7 studies, 58%) and medication management (11 studies, 92%) were predominant functions of HIT interventions among the studies retrieved. Telephone was a comparatively common mode of communication in 4 studies (33%), and data were communicated synchronously in 7 studies (58%).

Physiological monitoring was another common function of HIT interventions in studies involving patients with diabetes (9 studies, 75%). These studies usually involved manual (finger pricks) or continuous (subcutaneous sensor) blood glucose monitoring to provide a feedback mechanism for patients, caregivers, or clinicians to understand the behaviors that lead to hypo- or hyperglycemia. Additionally, continuous recording of blood glucose has been used to detect nighttime hypoglycemic episodes [38]. In two cases [33,34], data from portable insulin pumps were also monitored asynchronously. HIT interventions were used to transfer monitoring data to a caregiver’s mobile telephone (via short message service, ie, SMS) in two studies. Blood glucose data could usually be downloaded or uploaded and communicated to health care providers. In these cases, the HIT intervention sometimes also served a decision support function (3 studies, 25%).

Similar to the case with asthma, a goal of interventional strategies for pediatric patients with diabetes is to avoid the need for ED visits to address dangerous elevations in blood glucose. Frequency of ED visits was measured in two studies. Clinical outcomes (including glycosylated hemoglobin A1C, blood glucose, hypoglycemic episodes) were evaluated in 7 studies (58%). Among studies that focused on patients with type 1 diabetes, a comparatively high proportion were pilot studies (8 studies, 67%), while only 2 (17%) were development studies and 2 (17%) were evaluation studies.

In 7 studies (58%), interventions helped establish continuity of care. Diabetes studies also included several examples of HIT used to bridge geographical barriers to health care, another of the themes observed in the studies reported here (Table 7).

#### Special Needs

The term “special needs” describes the patient populations in 11 of the studies and has been defined as follows:

Children with special needs present a complex array of health care requirements that remain throughout their life span. These needs include chronic health disabilities (diabetes, epilepsy, cystic fibrosis), developmental and behavioral disorders (cerebral palsy, spina bifida, attention deficit hyperactivity disorder, mental retardation, autism), and traumatic injuries (traumatic brain injury, spinal cord injury) [45].

Effective diagnosis, care coordination, and case management for such patients can be complex and require specialist involvement [52].

Unlike studies that involved patients with asthma or type 1 diabetes, in studies that involved pediatric patients with special needs, communication was predominantly synchronous (10 studies, 91%) and videoconferencing was the most common mode of communication (7 studies, 64%). Synchronous communication (telephone or videoconference) between the hospital or specialist clinic and patients’ homes was generally used to improve continuity of care.

The most common functions among studies that involved children with special needs were consultation (8 studies, 73%) and diagnosis (7 studies, 64%). Telemedicine videoconferencing replaced in person examination, with virtual consultations reducing sometimes painful trips to the clinic and allowing for diagnosis, referrals, and recommendations on physical care [45-48,52]. Additionally, simultaneous communication among multiple providers, school staff, and the caregiver improved coordination of care with fewer physical trips to multiple clinics [45-48,52]. Such consultations also bridged geographical barriers to care (eg, for patients in rural areas) and increased access to specialists. Similar coordinated communication among multiple health care providers and caregivers was also achieved in a Swedish study in which modes of communication included Internet, email, and SMS [51]. One Italian study featured a portable device for monitoring physiological status and physical activities [53,54].

Studies in this category were mostly development phase (4 studies, 36%) or pilot phase (6 studies, 55%), while there were no evaluation studies and only one implementation study. In addition to patient outcomes, a larger than average proportion of these studies measured caregiver (100% of studies) and health care provider outcomes (9 studies, 83%) including satisfaction, feasibility, usability and usage. Some telemedicine studies conducted economic analyses.

#### Psychiatric Disorders

A recurring problem described in many of the 10 child psychiatry studies was the shortage of pediatric mental health specialists. Most of the studies featured telemedicine interventions, which have an established history in adult psychiatry and are considered suitable because much of the diagnosis and treatment in this setting is achieved by audiovisual communication [65]. Similar to studies involving children with special needs, the HIT technology used in studies that involved children with psychiatric disorders was predominantly synchronous in 8 studies (80%), with videoconference the principal mode of communication in 7 studies (70%). HIT was applied in 2 (20%) of studies to deliver cognitive behavioral therapy to patients with anxiety disorder [56,57].

Rather than coordinating care involving multiple providers, as is commonly seen with special needs, videoconferencing was used primarily for diagnosis in 7 studies (70%), for mental health therapy in 6 studies (60%), and for medication management in 4 studies (40%). A recurring theme was the use of HIT to bridge geographical barriers and the shortage of child mental health practitioners. Rural patients in the United States, Canada, and Australia represented the main population to receive telemedicine interventions (80% of studies).

Of these studies, 8 (80%) were pilot phase, and 2 (20%) were development phase. The purpose of pilot phase studies was often to evaluate satisfaction (9 studies, 90%) or to determine whether pediatric telepsychiatry was comparable with face-to-face treatment (eg, diagnostic agreement).

## Discussion

### Principal Results

We have observed how, in the health literature of HIT applications that facilitate communication among caregivers and health care providers, the pediatric diseases that are well represented are those characterized by high prevalence (asthma, type 1 diabetes), acute need caused by geographical barriers or other lack of health care provider access (psychiatric disorders, cardiac disorder, burns), or those requiring continuity of care in home or community settings (type 1 diabetes, special needs, cancer, complex health care needs post-discharge). Efforts to estimate the value of HIT interventions in these cases have included measurement of patient- or caregiver-important outcomes such as quality of life (21 studies, 20%) or clinical outcomes (33 studies, 32%), and evaluations of resource use that often comprise some degree of economic analysis (26 studies, 25%). Few studies, however, were capable of providing definitive evidence (ie, 17 studies, [16%] were RCTs, while only 11 [11%] qualified as evaluation studies). This is to be expected in research involving complex interventions, which is often constrained by methodological limitations and high cost.

### Implications

Several uses for scoping reviews as articulated by Anderson and colleagues [6] apply to the current study. Specifically, it has proved valuable “to map and make sense of the extent, range, and nature of research undertaken in a particular area,” and “to identify the strengths and weaknesses of the research base.” Others may also find this report useful “to identify gaps in research knowledge that need filling” and “to determine the value of undertaking further systematic reviews or empirical research.”

We suggest that opportunities exist to improve the utility of future development and evaluation work by focusing energies whenever possible on planning integrated programs of development, evaluation, and implementation as recommended by the MRC guidance for complex interventions [16]. Although the realities of some contexts can make this ideal impracticable, eight examples of researchers using phased approaches (featuring multiple related studies) to development and evaluation of their interventions were found here [8,10,18,21,25,30,65,66,77,78,92-94,102-105,108,109].

For this review, articles were considered to refer to the same study if they investigated the same intervention in the same set of patients. Accordingly, seven of the included studies corresponded to multiple publications. In all but one case, however, there was a lack of cross-reference between publications corresponding to the same study. Moreover, in two studies corresponding to five articles authored by the same study group, the same results were reported in multiple publications without any cross reference. More uniform use of study identifiers, as recommended in the CONSORT statement [124,125], may therefore be warranted to avoid multiple reporting in studies of HIT. Of the 104 studies included in this review, we found that only one [104] referred to CONSORT in its bibliography.

### Limitations

Scoping reviews are often characterized by the challenge of searching the literature for complex or ill-defined topics. Thus, unlike systematic reviews that typically have a narrower focus, it may be time-consuming and unrealistic to retrieve and screen all the relevant literature. As our purpose was to merely map the existing health literature on a complex topic and not estimate the effects of HIT interventions, our efforts to identify all eligible studies were limited in some respects. Consistent with our objective, we restricted our search to health databases, leaving out the engineering and computing literature. Also, we considered it unnecessary for our purpose to follow up on all of the many narrative reviews on HIT retrieved by our search. Due to this and the complexity of the topic, our study therefore cannot be considered an exhaustive accounting of the literature in this area. Nevertheless, the searches we designed were broad enough to expect that sensitivity, at least within the health literature, was moderately high. Supportive of this, the bibliographies of included articles yielded only three additional studies not detected by our search. Future reviewers focusing on more limited subsets of the literature than we have surveyed here will be able to employ more exhaustive search methods and may retrieve more articles than reported here.

### Conclusions

This study provides a map of the health literature on how HIT is being used and studied to facilitate care of pediatric patients with health conditions requiring follow-up and involving participation of both a caregiver and a health care provider. We have observed how HIT used for this purpose has been implemented differently in a range of disease settings, and how varying needs affect the function, form, and synchronicity of information transfer. Interventions have been repeatedly applied to improve continuity of care, address time constraints faced by health care providers, and bridge geographical barriers. Although a number of authors have followed a phased approach to development, evaluation, and implementation, a greater emphasis on methodological standards such as the MRC guidance for complex interventions would produce more fruitful programs of development and more useful evaluations in the future. This review will be especially helpful to those deciding on areas where further development or research into HIT in pediatric contexts may be warranted.

## Mutlimedia Appendix 1

Database searches

## Abbreviations

HIT: health information technology
ICT: information communication technology
IOM: Institute of Medicine
SMS: short message service
