# Supplementary material for: Health Information Technology to Facilitate Communication Involving Health Care Providers, Caregivers, and Pediatric Patients: A Scoping Review
Source: J Med Internet Res. 2010 Jun 18;12(2):e22. doi: 10.2196/jmir.1390 (PMC2956233; doi:10.2196/jmir.1390)
Supplement: Supplementary file 1 [file jmir_v12i2e22_app1.pdf]

Multimedia Appendix

Database Searches

MEDLINE search

#	Search History
1	(child: or adolescent or infan:).mp.
2	(online or on-line).tw.
3	(remote: adj2 consult:).tw.
4	(web page: or webpage:).tw.
5	(web-site: or website:).tw.
6	Answering Services/
7	cellular phone/
8	cyber:.tw.
9	discussion list:.tw.
10	e-bulletin board:.tw.
11	electron: bulletin board:.tw.
12	electron: discuss: board:.tw.
13	electron: mail:.tw.
14	electronic mail/
15	electronic:.tw.
16	e-mail:.tw.
17	email:.tw.
18	exp computer communication networks/
19	exp online systems/
20	exp computer-assisted instruction/
21	exp diagnosis, computer-assisted/
22	exp therapy, computer-assisted/
23	exp computers/
24	exp medical informatics/
25	exp computers, handheld/
26	exp decision making, computer-assisted/
27	exp electronics, medical/
28	exp internet/
29	exp microcomputers/
30	exp telecommunications/
31	exp user-computer interface/
32	(handheld adj2 computer:).tw.
33	(hand held adj2 computer:).tw.
34	information highway:.tw.
35	information superhighway:.tw.
36	information super highway:.tw.
37	instant messag:.tw.
38	text messag:.tw.
39	internet:.tw.
40	(irc and (internet: or online or on-line or chat: or relay)).tw.
41	list serv:.tw.
42	listserv:.tw.
43	mail: list:.tw.
44	messaging:.tw.
45	Microscopy, Video/
46	modems/

47	mobile phone:.tw.
48	mobilephone:.tw.
49	mobile telephone:.tw.
50	mobile telecom:.tw.
51	mobile communic:.tw.
52	newsgroup:.tw.
53	((pda or pdas) and (comput: or internet: or wireless:)).tw.
54	personal digital assistant:.tw.
55	pocket pc:.tw.
56	Radar/
57	Radio/
58	Remote Consultation/
59	Satellite Communications/
60	short messag:.tw.
61	(sms and (internet: or online or on-line or chat: or relay: or wireless:)).tw.
62	exp Software/
63	tele:.tw.
64	exp Telecommunications/
65	teleconfer:.tw.
66	tele-confer:.tw.
67	teleconsult:.tw.
68	tele-consult:.tw.
69	Telefacsimile/
70	(tele-home: or telehome:).tw.
71	telemed:.tw.
72	tele-med:.tw.
73	pocketpc:.tw.
74	Telemedicine/
75	exp Telephone/
76	usenet:.tw.
77	videoconfer:.tw.
78	video-confer:.tw.
79	Videoconferencing/
80	videophone:.tw.
81	virtual.tw.
82	web based:.tw.
83	webbased:.tw.
84	world wide web:.tw.
85	www.tw.
86	telestroke.mp.
87	tele-stroke:.mp.
88	Teleneurology.mp.
89	tele-neurology.mp.
90	telehealth:.mp.
91	tele-health:.mp.
92	tele-rehab:.mp.
93	teleservic:.mp.
94	tele-servic:.mp.
95	telerehab:.mp.
96	or/2-95
97	"delivery of health care"/ or delegation, professional/ or provider-sponsored organizations/ or health

	services accessibility/ or uncompensated care/
98	"Referral and Consultation"/
99	consult$.mp.
100	Organizational Innovation/
101	exp "diffusion of innovation"/ or technology transfer/
102	Practice Management, Medical/
103	workflow:.mp.
104	productivity.mp.
105	rural:.mp.
106	communit:.mp.
107	Primary Health Care/
108	northern territory/
109	health services, indigenous/
110	exp Primary Health Care/
111	exp Physicians, Family/
112	exp Family Practice/
113	exp Community medicine/
114	exp Group practice/
115	exp Physician's Practice Patterns/
116	exp Physicians' Offices/
117	exp Practice management, medical/
118	exp Regional medical programs/
119	exp ambulatory care/
120	exp outpatient clinics/
121	exp homes for the aged/
122	home nursing.mp. [mp=title, original title, abstract, name of substance word, subject heading word]
123	house calls/
124	house call:.mp.
125	housecall:.mp.
126	private practice/
127	Rural Health Services/
128	Hospitals, Rural/
129	Rural Health/
130	Rural Population/
131	northwest territories/ or nunavut/ or yukon territory/
132	Arctic Regions/
133	indians, north american/ or inuits/
134	outpatient:.mp.
135	out-patient:.mp.
136	(free-standing adj2 clinic?).mp.
137	(free-standing adj2 facilit:).mp.
138	ambulatory care facilities/
139	community health centers/
140	substance abuse treatment centers/
141	community mental health centers/
142	child guidance clinics/
143	maternal-child health centers/
144	outpatient clinics, hospital/
145	pain clinics/
146	surgicenters/
147	exp ambulatory care information systems/
148	general practice?.mp.
149	general practitioner?.mp.

150	home care services/
151	or/97-150
152	1 and 96 and 151
153	limit 152 to yr="1996 - 2008"
154	limit 153 to english
155	154 not (letter or editorial or news or comment).pt.

EMBASE search

#	Search History
1	(online or on-line).tw.
2	(remote: adj2 consult:).tw.
3	(web page: or webpage:).tw.
4	(web-site: or website:).tw.
5	answering service$.mp.
6	exp mass communication/
7	cyber:.tw.
8	discussion list:.tw.
9	e-bulletin board:.tw.
10	electron: bulletin board:.tw.
11	electron: discuss: board:.tw.
12	electron: mail:.tw.
13	electronic:.tw.
14	e-mail:.tw.
15	email:.tw.
16	exp information system/
17	(computer-assisted learning or computer-assisted instruction$ or computer-assisted education or computer assisted learning or computer assisted instruction$ or computer assisted education).tw.
18	computer assisted diagnosis/
19	exp computer assisted therapy/
20	computer/ or microcomputer/ or personal digital assistant/
21	medical informatics/
22	medical electronic$.tw.
23	computer interface/
24	(handheld adj2 computer:).tw.
25	(hand held adj2 computer:).tw.
26	information highway:.tw.
27	information superhighway:.tw.
28	information super highway:.tw.
29	instant messag:.tw.
30	text messag:.tw.
31	internet:.tw.
32	(irc and (internet: or online or on-line or chat: or relay)).tw.
33	list serv:.tw.
34	listserv:.tw.
35	mail: list:.tw.
36	messaging:.tw.
37	mobile phone:.tw.
38	mobilephone:.tw.
39	mobile telephone:.tw.

40	mobile telecom:.tw.
41	mobile communic:.tw.
42	newsgroup:.tw.
43	((pda or pdas) and (comput: or internet: or wireless:)).tw.
44	personal digital assistant:.tw.
45	pocket pc:.tw.
46	pocketpc:.tw.
47	short messag:.tw.
48	(sms and (internet: or online or on-line or chat: or relay: or wireless:)).tw.
49	exp computer program/
50	tele:.tw.
51	teleconfer:.tw.
52	tele-confer:.tw.
53	teleconsult:.tw.
54	tele-consult:.tw.
55	(tele-home: or telehome:).tw.
56	telemed:.tw.
57	tele-med:.tw.
58	usenet:.tw.
59	videoconfer:.tw.
60	video-confer:.tw.
61	videophone:.tw.
62	virtual.tw.
63	web based:.tw.
64	webbased:.tw.
65	world wide web:.tw.
66	www.tw.
67	telestroke.mp.
68	tele-stroke:.mp.
69	Teleneurology.mp.
70	tele-neurology.mp.
71	telehealth:.mp.
72	tele-health:.mp.
73	telerehab:.mp.
74	tele-rehab:.mp.
75	teleservic:.mp.
76	tele-servic:.mp.
77	or/1-76
78	exp Health Care Delivery/ or Professional Delegation/
79	exp Patient Referral/
80	consult$.mp.
81	exp Organization/
82	(diffusion of innovation or "technology transfer").tw.
83	management/
84	workflow:.mp.
85	productivity.mp.
86	rural:.mp.
87	communit:.mp.
88	indigenous health services.mp.
89	exp general practitioner/
90	exp community medicine/
91	Physician's Practice Patterns.mp.

92	Physicians' Offices.mp. or Health Care Facility/
93	exp Home for the Aged/
94	exp Professional Practice/
95	house call:.mp.
96	housecall:.mp.
97	exp Rural Health Care/
98	exp Rural Health Nursing/
99	exp Rural Population/
100	(northwest territories or nunavut or yukon).mp.
101	exp Arctic/
102	exp american indian/ or exp eskimo/
103	outpatient:.mp.
104	out-patient:.mp.
105	(free-standing adj2 clinic?).mp.
106	(free-standing adj2 facilit:).mp.
107	exp Health Center/
108	exp Drug Dependence Treatment/
109	exp Community Mental Health Center/
110	child guidance clinic$.mp.
111	exp Pain Clinic/
112	exp Hospital Information System/
113	general practice?.mp.
114	general practitioner?.mp.
115	medical care/ or clinical decision making/ or doctor patient relation/ or general practice/ or medical decision making/ or primary medical care/ or private practice/
116	patient care/ or "bladder and bowel management"/ or breast care/ or case finding/ or case management/ or catheter care/ or cultural safety/ or eye care/ or foot care/ or gynecologic care/ or holistic care/ or patient assessment/ or patient care planning/ or patient decision making/ or patient positioning/ or patient scheduling/ or perineal care/ or peroperative care/ or postanesthesia care/ or rehabilitation care/ or urological care/ or exp patient monitoring/
117	or/78-116
118	77 and 117
119	child:.mp.
120	adolescent.mp.
121	infan:.mp.
122	exp pediatrics/
123	or/119-122
124	118 and 123
125	limit 124 to yr="1996 - 2008"
126	limit 125 to english
127	126 not (letter or editorial or news or comment).pt.

CINAHL search

#	Search History
1	(online or on-line).tw.
2	(remote: adj2 consult:).tw.
3	(web page: or webpage:).tw.

4	(web-site: or website:).tw.
5	answering service$.mp.
6	exp informatics/
7	cyber:.tw.
8	discussion list:.tw.
9	e-bulletin board:.tw.
10	electron: bulletin board:.tw.
11	electron: discuss: board:.tw.
12	electron: mail:.tw.
13	electronic:.tw.
14	e-mail:.tw.
15	email:.tw.
16	exp teaching methods/
17	(handheld adj2 computer:).tw.
18	(hand held adj2 computer:).tw.
19	information highway:.tw.
20	information superhighway:.tw.
21	information super highway:.tw.
22	instant messag:.tw.
23	text messag:.tw.
24	internet:.tw.
25	(irc and (internet: or online or on-line or chat: or relay)).tw.
26	list serv:.tw.
27	listserv:.tw.
28	mail: list:.tw.
29	messaging:.tw.
30	(video microscop$ or video-microsop$).tw.
31	mobile phone:.tw.
32	mobilephone:.tw.
33	mobile telephone:.tw.
34	mobile telecom:.tw.
35	mobile communic:.tw.
36	newsgroup:.tw.
37	((pda or pdas) and (comput: or internet: or wireless:)).tw.
38	personal digital assistant:.tw.
39	pocket pc:.tw.
40	pocketpc:.tw.
41	radar.tw.
42	short messag:.tw.
43	(sms and (internet: or online or on-line or chat: or relay: or wireless:)).tw.
44	tele:.tw.
45	exp Telecommunications/
46	teleconfer:.tw.
47	tele-confer:.tw.
48	teleconsult:.tw.
49	tele-consult:.tw.
50	(tele-home: or telehome:).tw.
51	telemed:.tw.
52	tele-med:.tw.
53	usenet:.tw.
54	videoconfer:.tw.
55	video-confer:.tw.
56	videophone:.tw.

57	virtual.tw.
58	web based:.tw.
59	webbased:.tw.
60	world wide web:.tw.
61	www.tw.
62	telestroke.mp.
63	tele-stroke:.mp.
64	Teleneurology.mp.
65	tele-neurology.mp.
66	telehealth:.mp.
67	tele-health:.mp.
68	telerehab:.mp.
69	tele-rehab:.mp.
70	teleservic:.mp.
71	tele-servic:.mp.
72	or/1-71
73	exp Health Care Delivery/ or professional delegation.mp. or exp Provider-Sponsored Organizations/ or exp Health Services Accessibility/ or exp Uncompensated Care/
74	exp "Referral and Consultation"/
75	consult$.mp.
76	exp Organizational Culture/ or exp Organizational Change/
77	exp "Diffusion of Innovation"/ or exp Product Evaluation/ or exp Product Development/ or exp Equipment Design/ or exp Assistive Technology/ or technology transfer.mp. or exp Assistive Technology Devices/
78	exp Private Practice Management/
79	workflow:.mp.
80	productivity.mp.
81	rural:.mp.
82	communit:.mp.
83	exp Northern Territory/
84	exp Health Services, Indigenous/
85	exp Physicians, Family/
86	exp Family Practice/
87	exp Community Health Services/
88	exp Group Practice/
89	Physician's Practice Patterns.mp.
90	exp Practitioner's Office/
91	Regional medical programs.mp.
92	exp Ambulatory Care/
93	exp Outpatient Service/
94	exp Nursing Homes/
95	exp Home Nursing/
96	exp Home Health Care/
97	house call:.mp.
98	housecall:.mp.
99	exp Private Practice/
100	exp Rural Health Services/
101	exp Hospitals, Rural/
102	exp Rural Health/
103	exp Rural Areas/
104	exp northwest territories/ or exp nunavut/ or exp yukon

	territory/
105	exp arctic regions/
106	exp eskimos/ or exp native americans/
107	outpatient:.mp.
108	out-patient:.mp.
109	(free-standing adj2 clinic?).mp.
110	(free-standing adj2 facilit:).mp.
111	exp Ambulatory Care Facilities/
112	exp Community Health Centers/
113	exp "Substance Use Rehabilitation Programs"/
114	exp Community Mental Health Services/
115	child guidance clinics.mp.
116	maternal-child health centers.mp.
117	exp ambulatory care information systems/
118	general practice?.mp.
119	general practitioner?.mp.
120	or/74-119
121	73 and 120
122	child:.mp.
123	adolescent.mp.
124	infan:.mp.
125	or/122-124
126	121 and 125
127	limit 126 to yr="1996 - 2008"
128	limit 127 to english
129	128 not (letter or editorial or news or comment).pt.
